# Properties Analysis of Asphalt Binders Containing Bayer Red Mud

**DOI:** 10.3390/ma13051122

**Published:** 2020-03-03

**Authors:** Liyang Yao, Wenying Gao, Xianwei Ma, Hao Fu

**Affiliations:** School of Civil and Transportation Engineering, Henan University of Urban Construction, Pingdingshan 467036, China; 20130121@hncj.edu.cn (W.G.); maxianwei9733@163.com (X.M.); fuhao9733@163.com (H.F.)

**Keywords:** asphalt binder, Bayer red mud, limestone, filler, performance

## Abstract

In this work, related performances of asphalt binders with Bayer red mud powder (RMP) were studied. RMP replaced the traditional limestone powder (LSP) as a filler in asphalt binder. The replacement rates were 0%, 25%, 50%, 75%, and 100%, respectively. In this study, seven F/A (filler-to-asphalt, weight/weight) ratios for each of the fillers were selected: 0.3, 0.6, 0.9, 1.2, 1.5, 1.8, and 2.1. Penetration, softening point, rotational viscosity (RV), dynamic shear rheometry (DSR), and bending beam rheometry (BBR) tests were used to evaluate the properties of the asphalt binder. Penetration into the asphalt binder decreases linearly with increasing F/A ratio. Moreover, penetration of binder with RMP is lower than that of asphalt binder with LSP (RMP0), and among the five fillers tested, RMP100 showed most significant influence on penetration of the asphalt binder. The addition of RMP increases the softening point of the binder. DSR results show that the improvement in the high temperature performance is most significant after replacing 75% of the LSP with Bayer RMP. BBR results show that with increasing substitution of RMP for LSP, the creep stiffness (S) increased while the rate of change of S (m-value) declined. The low temperature performance of every asphalt binder was not enough to meet the Superpave requirements. In order to meet the Superpave requirements for S and m-values, the maximum F/A ratios of the five replacements corresponding to the fillers with 0%, 25%, 50%, 75%, and 100% RMP, were 1.3, 1.2, 1.1, 1.0 and 0.9, respectively. At 135 °C, rotational viscosity showed that RMP75 and RMP100 with a maximum F/A ratio of 1.1 are the best choices for asphalt binders, considering economic and construction requirements.

## 1. Introduction

Red mud is a reddish brown colored alkaline solid waste from the alumina refining of bauxite ore [1]. Bayer red mud is the industrial waste slag produced by the Bayer process in the alumina plant. A ton of alumina products generates about 0.8–1.5 tons of red mud according to the grade of bauxite ores and operating conditions [2]. China is the largest producer of alumina and red mud in the world [3]. About 30 million tons of red mud are generated yearly in China, and nearly 4 billion tons of red mud has now accumulated [4].

The treatment and utilization of red mud has been a huge challenge for the governments and alumina industry around the world, especially in China. In recent years, investigators around the world have researched the treatment and utilization of red mud. These investigations mostly focused on utilizing the red mud to produce building materials (such as bricks, novel inorganic polymer paving blocks, cement, concrete, glass, and ceramics) [5,6,7,8,9,10,11,12], recover valuable elements [13,14], purify gas [15], treat water [16], and improve soil [17]. The building materials industry has been very interested in the treatment and utilization of red mud, because it can be cost effective. However, the high alkalinity (pH 10–12.5) of red mud results in difficulties when used as raw materials for buildings. Fortunately, asphalt in the asphalt mixture is slightly acidic and the alkalinity of the red mud is beneficial to improving the adhesion between the asphalt and the aggregate, according to chemisorption theory.

Mineral fillers are an important part of asphalt mixtures. It has a great influence on the cohesive performance of asphalt binders [18,19]. Traditionally, naturally alkaline limestone powder (LSP) is used as the most common mineral filler in asphalt mixtures. However, limestone is a natural resource and is being exhausted with the increased development of the cement and construction industry in China [20]. The production of LSP causes a large amount of dust which will pollute the environment. Therefore, many investigators have been studying waste materials to replace the LSP for use as asphalt binders and in asphalt mixtures. Researchers have successfully found several waste materials (such as steel slag, recycled red brick powder, rice husk ash, coal waste powder, fly ash, and waste lime) which can replace LSP as fillers in asphalt mixtures [21,22,23,24,25,26]. The utilization of these waste materials as fillers in asphalt mixtures enlarges their application fields and can make them valuable commodities. Even with these new materials, the requirements needed by the rapid development of the construction and traffic infrastructure in China cannot be met. These materials are not evenly distributed throughout China and their high processing costs also limit their use. Also, only some of these new materials are well suited for use in asphalt mixtures. Many of the materials are not alkaline which adversely affects the adhesive properties between the asphalt and the aggregate. Therefore, additional materials are urgently needed to supplement them.

Red mud, as a waste alkaline material, not only occupies a large amount of land resources, but also results in serious environmental pollution [27]. Red mud has been widely used as a building material in the field of construction engineering. However, the application of red mud in the field of road engineering has only been reported in the literature a few times [28,29], especially in asphalt binder and asphalt mixtures. This paper mainly investigates the performances of asphalt binders with red mud powder (RMP) obtained by special treatment, to replace LSP either partially or completely as a filler.

In order to study the performance of asphalt binder adding RMP, various tests were performed, including penetration, softening point, rotational viscosity (RV), dynamic shear rheometry (DSR), and bending beam rheometry (BBR). Comparisons with asphalt binders prepared using the conventional LSP are also made. RV, DSR, and BBR are the tests used to determine if asphalt binders can be used in Superpave applications. Superpave is one of the asphalt outcomes of the Strategic Highway Research Program (SHRP) of the United States between 1987 and 1992. Superpave presented new tests and standards for asphalt binders, which provides a basis for the of study asphalt binders in this paper.

## 2. Materials and Methods

### 2.1. Materials

In this study, the AH-70 base asphalt was supplied by the Koch Asphalt Company (HongKong, China). It was used in the preparation of asphalt binder. As per JTG E20-2011 [30] of China, the penetration (0.1 mm at 25 °C, 100 g, and 5 s), the softening point and the ductility (at 15 °C and 5 cm/min) of the base asphalt is 69 (0.1 mm), 46.7 °C, 163 cm, respectively.

**Figure 1:**
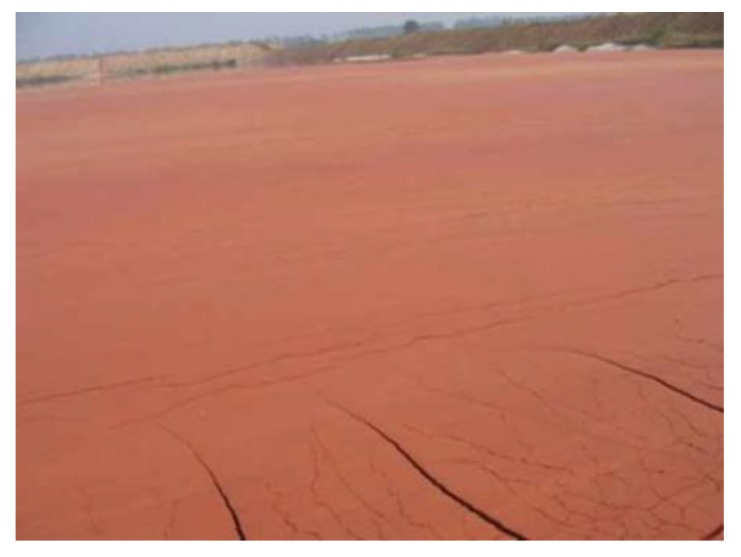
Red mud landfill.

**Figure 2:**
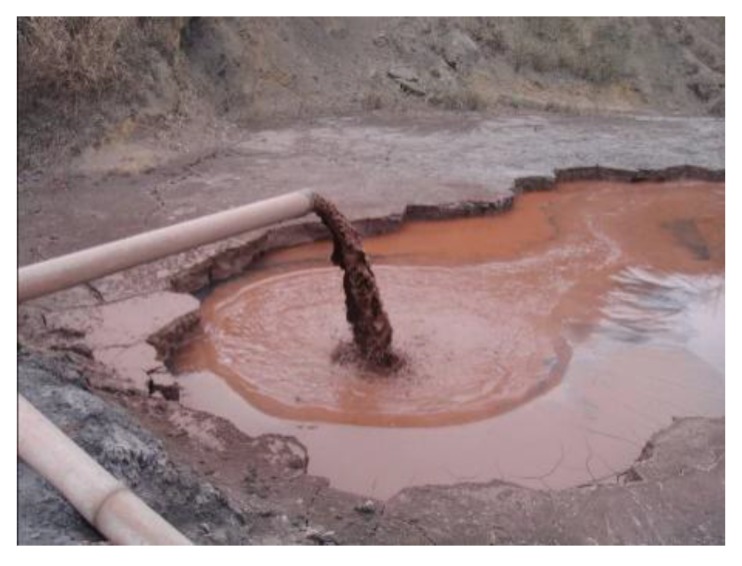
Red mud discharge.

**Figure 3:**
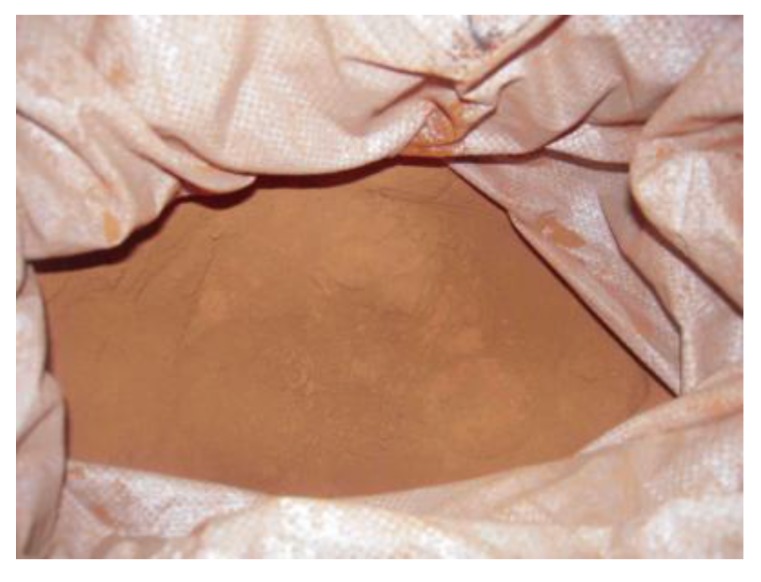
Red mud powder (RMP).

Natural LSP, which will be used as a reference, was obtained from Baofeng County, which is located in the Henan Province of Central China. The Bayer red mud used in this paper came from the Jiaozuo Zhongzhou alumina refinery in the Henan province of Central China. It is the largest aluminum plant in Henan province and produces about 2 million tons of red mud annually. At this moment, 19 million tons of red mud have already been stockpiled over an area which covers more than 1300 acres, which is depicted in Figure 1; Figure 2. The red mud was dried at 105 °C for 5 h, and then crushed by a jaw crusher and ground by ball mill for 30 min to obtain the RMP, as shown in Figure 3. The basic performances of LSP and Bayer RMP are given in Table 1. The chemical compositions determined by X-ray fluorescence spectroscopy (XRF) (Panalytical Axios, Almelo, Holland) can be found in Table 2. Table 2 shows that the major oxide component of LSP is CaO. CaO is an alkaline compound which affects the bond between the weakly acidic asphalt and the aggregate. However, the concentration of CaO of Bayer RMP is less than in LSP. Therefore, the combination of Bayer RMP and LSP in asphalt binders can help improve the adhesive properties between the asphalt and the aggregate compared to RMP by itself. This may be because the larger amounts of CaO in the LSP can make up for the CaO deficiency in the Bayer RMP.

### 2.2. Preparation of the Asphalt Binder

In this study, five types of filler were used to prepare the asphalt binder. These fillers were composed of 0%, 25%, 50%, 75%, and 100% RMP instead of LSP respectively. Filler combinations are given in Table 3. For these fillers, seven different filler-to-asphalt (F/A) ratios were studied: 0.3, 0.6, 0.9, 1.2, 1.5, 1.8 and 2.1, respectively.

During the preparation of the asphalt binder, the base asphalt was heated in the mixing container inside the oven at 140 °C for 60 min until melt. According to the chosen F/A, a certain quantity of the filler heated at 140 °C was mixed with the base asphalt and stirred for 20 min at a speed of 800 rpm, until an even mixture was obtained. The asphalt binders were kept at room temperature for the subsequent performance tests.

### 2.3. Methods

In this work, the main test methods of the asphalt binder included penetration, softening point, rotational viscosity, dynamic shear rheometry (DSR) and bending beam rheometry (BBR). Softening point, rotational viscosity (RV), and penetration are the basic performance parameters of road asphalt, which have been applied to evaluating asphalt binder [31,32]. Penetration (0.1 mm at 100 g and 5 s) at 25 °C, softening point (ring and ball), and rotational viscosity at 135 °C of the asphalt binder were tested according to JTG E20-2011 [30] of China. Rotational viscosity was measured using a rotational viscometer (NDJ-1C, CHANGJI, Shanghai, China). The asphalt sample was poured into a chamber holder and then inserted into the RV chamber to achieve the desired temperature of 135 °C. Viscosity of the asphalt binder was determined at 135 °C. A cylindrical spindle was submerged in the chamber and was rotated at a speed of 20 rpm.

In this study, dynamic shear rheometry (DSR) (DHR-I, TA, New Castle, DE, USA) and bending beam rheometry (BBR) (ATS, PA, USA) were applied to characterize the rheological performances of all asphalt binders. The dynamic shear rheometry (DSR) test was performed at 60 °C at a fixed frequency of 10 rad/s. Parallel plates with a diameter of 25 mm were used to prepare binder samples with the thickness of 2 mm. The asphalt binders were heated until they became sufficiently fluid enough to be poured into silicone molds to obtain the DSR test samples. The test was carried out with 25 mm diameter, 1 mm gap geometry at 60 °C. Through the DSR test, the complex shear modulus (G*), phase angle (δ), and rutting factor (G*/sin δ) of the asphalt binder were analyzed in detail. The bending beam rheometry (BBR) test was performed to determine the creep response of the asphalt binder at −12 °C and a loading time of 240 s. The size of the specimen was 127 × 6.35 × 12.7 mm. During the test, a beam of the specimen was submerged in a constant temperature bath and kept for 60 min. A constant load of about 100 g was applied after preloading on to the rectangular beam. The beam was supported by stainless steel half rounds on both ends. By measuring the deflection of the center of specimen continuously, the creep stiffness (S) and the rate of change of creep stiffness (m-value) of the asphalt binders were obtained.

## 3. Results and Discussion

### 3.1. Penetration

**Figure 4:**
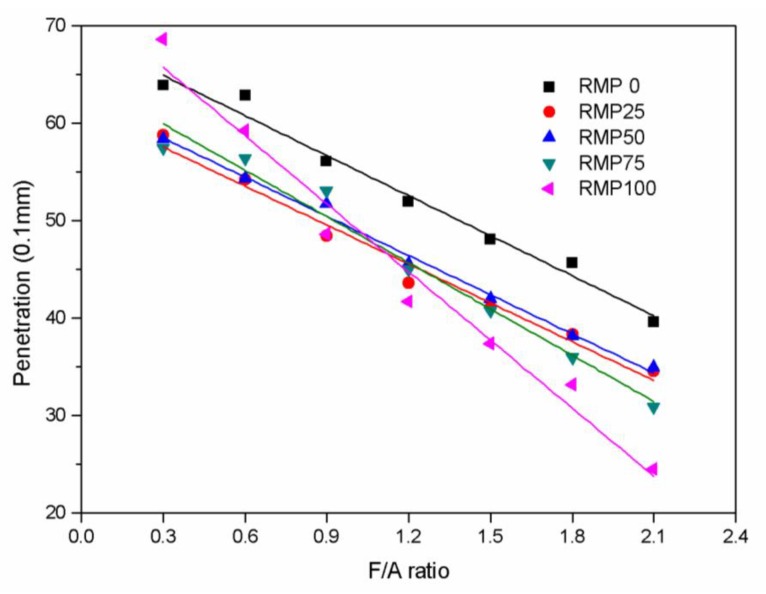
Penetration versus F/A ratio for the five kinds of fillers.

The values of the penetration versus the F/A ratio for each kind of asphalt binder are plotted in Figure 4. Figure 4 shows that the four asphalt binders containing RMP have smaller penetrations than RMP0 at the same F/A ratio, because the strong alkalinity of the RMP causes the asphalt binder to become more viscous. For these asphalt binders, penetration linearly decreases with increasing F/A ratio. Furthermore, the penetration of RMP100 asphalt binder drops faster with the F/A ratio than the other four, which shows that RMP100 has greater effects on the asphalt than the other four fillers. Table 4 shows the linear function relationship between the penetration value and F/A ratio for all of the asphalt binders, and all of the correlation coefficients are over 0.97.

### 3.2. Softening Point

**Figure 5:**
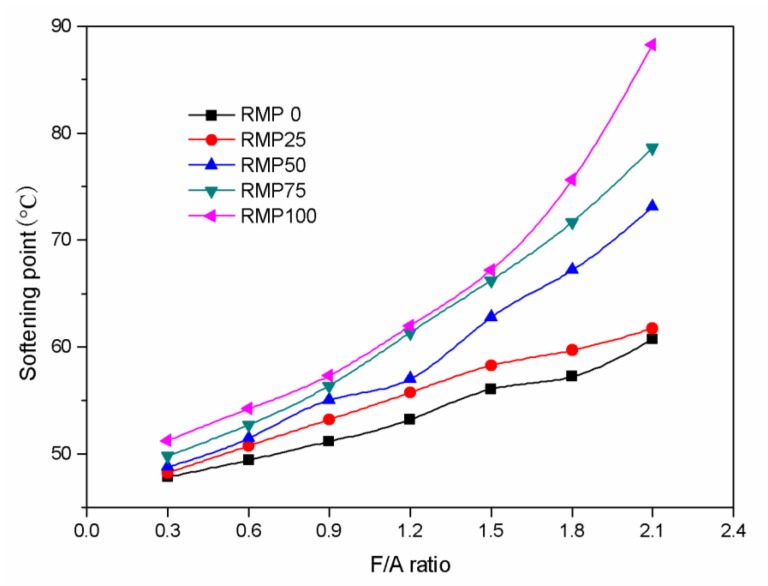
Softening point versus F/A ratio for the five different kinds of fillers.

The relationship between the softening point and F/A ratio is presented in Figure 5. The softening point of the five different kinds of asphalt binder increases markedly with increasing F/A ratio. The softening point of RMP100 asphalt binder is the highest and LSP (RMP0) asphalt binder is the lowest at the same F/A ratio. Furthermore, with the increase of RMP and the concurrent decrease of LSP in the filler, the softening point of the asphalt binder rises markedly. This shows that the influence of RMP on softening point of asphalt binder is significant. This may be due to the fact that the strong alkalinity of RMP results in more structural asphalt, which makes asphalt binder more viscous.

### 3.3. Rotational Viscosity

**Figure 6:**
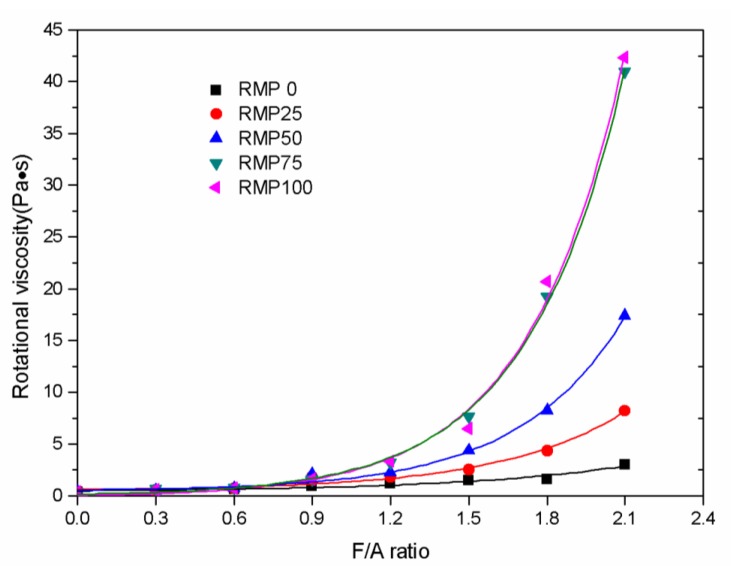
Rotational viscosity (RV) at 135 °C versus F/A ratio for the five different kinds of fillers.

The rotational viscosity (RV) value versus the F/A ratio for the asphalt binders in this study at 135 °C is plotted in Figure 6. From Figure 6 we can see that the rotational viscosity value rises exponentially as the F/A ratio rises for all asphalt binders. An exponential function is the best way to describe the relationship between the rotational viscosity values and the F/A ratio and the best fit functions are shown in Table 5. Figure 6 also shows that as RMP is substituted for LSP from 0% to 25% to 50% to 75% to 100%, the rotational viscosity of asphalt binders increases significantly, even when the F/A ratio is the same. The viscosity values were found to increase more at higher F/A ratios. Higher viscosity values for the asphalt binder in hot-mix asphalt (HMA) are important for rutting resistance and permanent deformation resistance of the finished asphalt pavement. However, excessive viscosity is detrimental to the construction of the asphalt mixture and the low temperature cracking resistance of asphalt pavement. Therefore, the rotational viscosity at 135 °C has to be less than the 3 Pa·s maximum value requirement for use in Superpave. Further, the temperature of 135 °C represents the most common mixing temperature for hot-mixed asphalt (HMA) mixtures. At 135 °C, the rotational viscosity value of 3 Pa·s occurs for RMP0, RMP25, RMP50, RMP75, and RMP100 at the F/A ratios of 2.0, 1.5, 1.2, 1.1, and 1.1, respectively. Economics and usability suggest that RMP75 and RMP100 with a maximum F/A ratio of 1.1 are the best choices for use in asphalt binders.

### 3.4. Dynamic Shear Rheometry (DSR)

**Figure 7:**
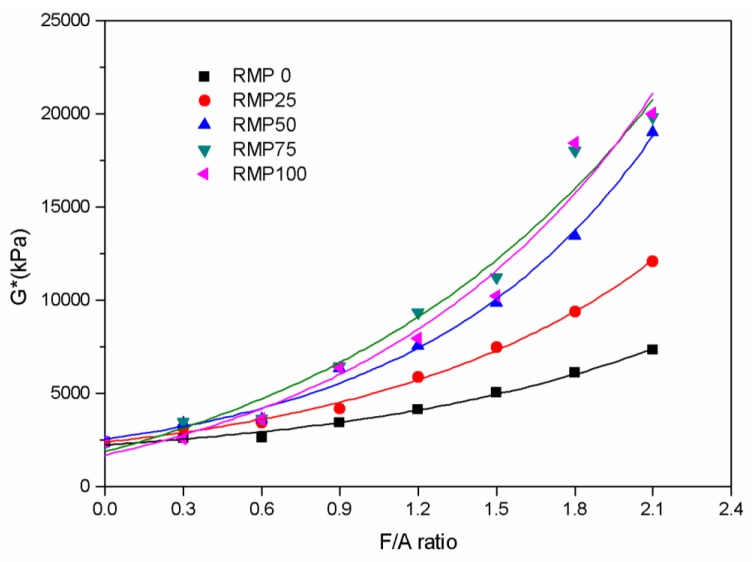
G* values versus F/A ratio for the five different kinds of fillers.

DSR can evaluate the high temperature properties of the asphalt binders. Through this test, two parameters, G* (complex shear modulus) and δ (the phase angle), were directly obtained for all asphalt binder specimens at 60 °C. G* values obtained for the five different fillers are plotted against the F/A ratio in Figure 7. The G* values rise exponentially as the F/A ratio rises for all of the asphalt binders in this study. Exponential functions were found to be the best way to describe the relationship between the G* values and F/A ratio. Table 6 shows the best fit exponential functions for the G* values and the F/A ratio for every kind of asphalt binder and the correlation coefficients are all higher than 0.97. The results show that the F/A ratio has a marked influence on G*. G* values reflect the total resistance in the deformation of asphalt binders. At a given temperature, when the G* value increases, the total resistance of asphalt binder deformation becomes larger. Figure 7 shows that the G* values of the asphalt binders are noticeably different with the amount of RMP in the filler, at the same F/A ratio. When the ratio of RMP in the binder increases, the G* value of the asphalt binder is also significantly improved. For every asphalt binder in this study, the G* values of the LSP asphalt binder is the smallest at the same F/A ratio. When the F/A ratio is not more than 1.5, the G* value of the binder with RMP75 filler is the highest, which means that their resistance to deformation is the strongest. When the F/A ratio is greater than 1.5, the resistance to deformation of the binder with RMP100 filler is the strongest.

**Figure 8:**
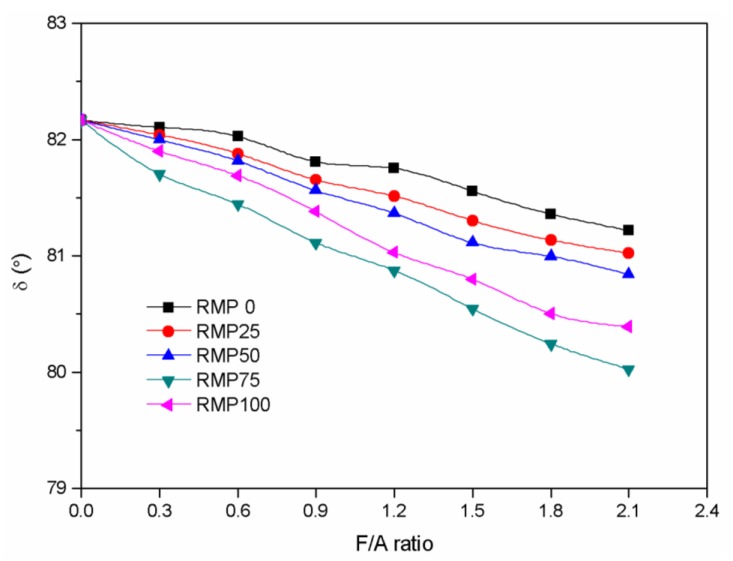
Phase angle (δ) versus F/A ratio for the five different kinds of fillers.

The phase angle is the ratio of elastic component (recoverable part of deformation) and viscous component (unrecoverable part of deformation) in asphalt binder. The greater the phase angle is, the more viscous component in the asphalt binder, which leads to a decrease in the high temperature performance of the asphalt mixture, such as rutting resistance. The smaller the phase angle is, the more elastic component in the asphalt binder, leading to stronger rutting resistance at high temperatures. Variations of the phase angle (δ) versus the F/A ratio for the five asphalt binders at 60 °C in this study are depicted in Figure 8. The phase angle decreased gradually as the F/A ratio increased for every asphalt binder tested. Compared with the other four kinds of asphalt binders, the phase angle of the RMP75 asphalt binder decreased the most significantly. The phase angle of the RMP0 asphalt binder had the smallest drop over the range of F/A ratios. This shows that RMP improves the high temperature properties of the asphalt binder, when added as a filler. When the F/A ratio is the same, increasing the amount of RMP leads to lower phase angles and better high temperature properties of the asphalt binder. The asphalt binder with RMP75 is found to be better than the others.

**Figure 9:**
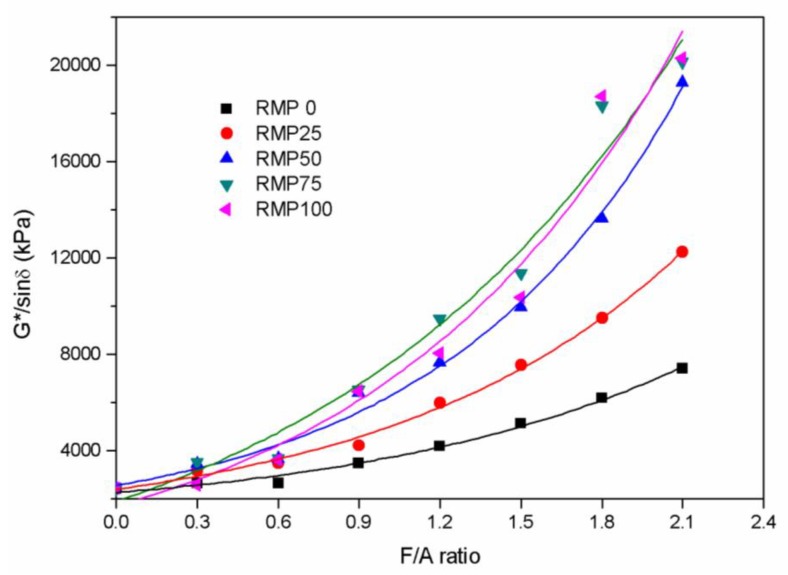
G*/sin(δ) versus F/A ratio for the five different kinds of fillers.

Rutting is a form of high temperature asphalt pavement damage. Asphalt binders have a significant influence on the rutting resistance of asphalt mixture. In the Superpave specification, G*/sin(δ), which is known as the rutting factor, is considered a high temperature evaluation index of asphalt binders. The greater the rutting factor, the stronger the resistance to rutting. G*/sin(δ) values were plotted against the F/A ratio in Figure 9. The best functional fit for the rutting factor versus the F/A ratio is also an exponential function. The relationship between the rutting factor and the F/A ratio is very similar to the relationship between the G* values and the F/A ratio. Table 7 shows that the correlation coefficients (R^2^) for the five models are all greater than 0.97. This shows that the rutting resistance of the asphalt binder rises with increasing F/A ratio. Additionally, the F/A ratio has the most prominent effect on the RMP75 asphalt binder and the smallest effect on the LSP asphalt binder among the five different asphalt binders. This indicates that the addition of RMP to the filler is a good way to improve the high temperature performance of asphalt binders.

### 3.5. Bending Beam Rheometry (BBR)

Bending beam rheometry (BBR) was used in this study to evaluate the low temperature rheological properties of asphalt binders with different fillers. BBR tests apply a specific load and show the creep stiffness (S) and the rate of change of the creep stiffness (m-value) of asphalt binders. S reflects the crack resistance of asphalt binders at low temperature. The higher the creep stiffness value, the worse the low temperature performance. The m-value reflects the relaxation performance of an asphalt binder at low temperature. The greater the m-value, the stronger the relaxation ability. The requirements of Superpave state that the S value should not be greater than 300 MPa and the m-value should not be less than 0.3 at −12 °C.

**Figure 10:**
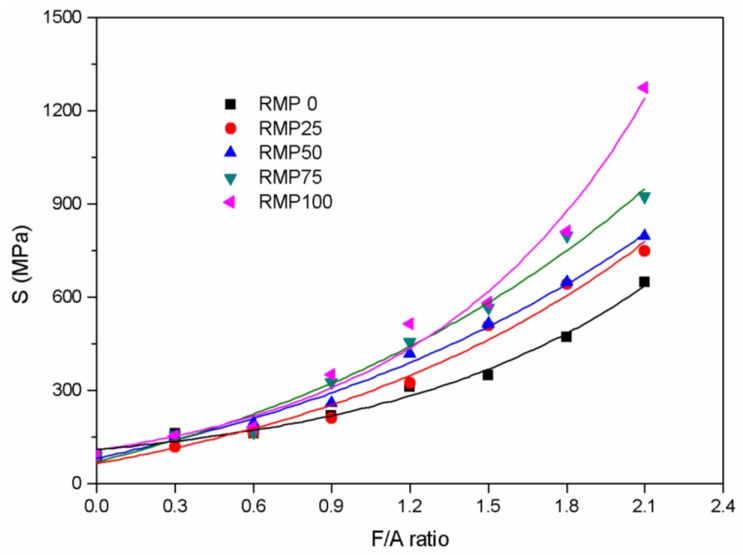
Creep stiffness (S) values versus F/A ratio by for the five different kinds of fillers.

Figure 10 shows the relationship between S and the F/A ratio for the five kinds of asphalt binders. The function which best describes the relationship between S and the F/A ratio is the exponential function with correlation coefficients (R^2^) greater than 0.97 shown in Table 8. This shows that the F/A ratio has a significant influence on S. However, the influence of lower F/A ratios on the creep stiffness of asphalt binder is not as significant, when compared with higher F/A ratios. When the F/A ratio is greater than 0.9, the S values of every asphalt binder increases significantly.

Figure 10 shows that with increased amounts of RMP in the filler, the rate of change of the S values increases gradually as the F/A ratio increases. The m-value of the RMP asphalt binder is the highest, while the LSP asphalt binder is the smallest. This showed that the influence of the RMP on creep stiffness is more significant than that of LSP. The higher the amount of RMP replacement, the more obvious the change of S as the F/A ratio increases. The five different asphalt binders met the requirement that the creep stiffness must not be greater than 300 MPa, and the maximum F/A ratios are 1.3, 1.2, 1.1, 1.0, and 0.9, respectively, for LSP (RMP0), RMP25, RMP50, RMP75, and RMP100.

**Figure 11:**
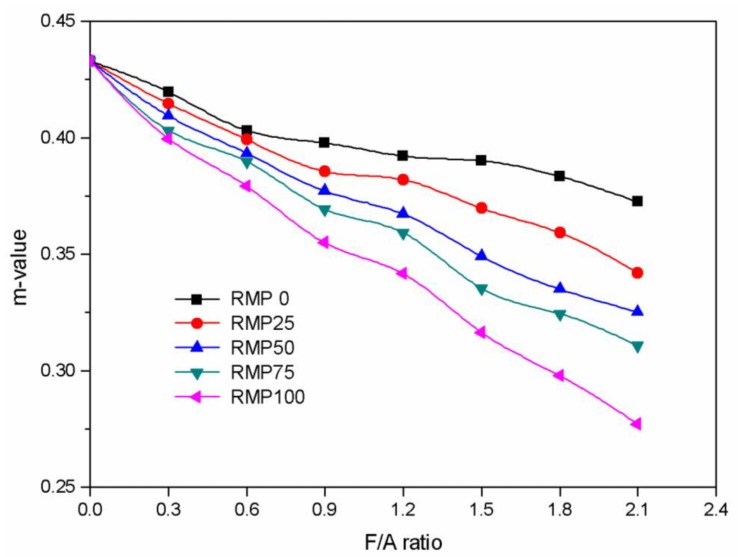
m-value versus F/A ratio for the five different kinds of fillers.

The effects of the F/A ratio on the m-value of the asphalt binders can be seen in Figure 11. For all of the asphalt binders, the m-values decreased gradually as the F/A ratio increased. Compared with the asphalt binder without filler (F/A ratio = 0), the LSP (RMP0), RMP25, RMP50, RMP75, and RMP100 asphalt binders m-values were reduced by 13.94%, 20.99%, 24.95%, 28.23%, and 36.06%, respectively. Also, increasing the amount of RMP in the filler leads to greater reductions in the m-values for the binders. This shows that the RMP has a stronger effect on m-value than LSP. Except for those binders with F/A ratios of 1.8 and 2.1, all of the asphalt binders tested with RMP meet the Superpave m-value requirement of not being less than 0.3 at low temperature (−12 °C).

## 4. Conclusions

This study evaluated the performance of asphalt binders using RMP as a filler, instead of the commonly used LSP. Based on the results of the studies performed, the following main conclusions can be given:

(1) In the Bayer red mud, Fe_2_O_3_, Al_2_O_3_, and SiO_2_ were the dominant components accounting for nearly 67% of the total weight. Na_2_O was detected in the Bayer red mud samples and contributes a strong alkaline effect, with a concentration of 3.21%. The higher Na_2_O concentration indicates why the red mud powders have higher PH values in comparison with more traditional natural mineral fillers. Moreover, this alkalinity may be beneficial in improving the adhesion between the asphalt and aggregates, due to the weak acidity of the asphalt.
(2) As the F/A ratio increases, the softening point increases gradually, and the penetration decreases linearly. The effects of RMP on the softening point and the penetration is more prominent than LSP.
(3) Exponential functions provide the best descriptions of the relationship between the rotational viscosity and the F/A ratio. The higher the amount of RMP in the binder, the more noticeably the rotational viscosity changes, and the smaller the F/A ratio which corresponds to the Superpave requirement of 3 Pa·s. For RMP100 asphalt binder, the F/A ratio that meets this requirement is 1.2.
(4) G* and G*/sin(δ) have an exponential relationship with the F/A ratio. RMP75 asphalt binder has the highest proportion of elastic component, because δ is the smallest. Moreover, the deformation resistance and the rutting resistance of the RMP75 asphalt binder at high temperature are the strongest in this work. LSP (RMP0) asphalt binder is the opposite, with the weakest deformation and rutting resistance.
(5) As the F/A ratio increases, the low temperature properties (S and m-values) of the asphalt binders decrease gradually. With increased substitution of RMP for LSP, the low temperature properties decrease more significantly. In order to meet the Superpave requirements, S values need to be less than 300 MPa, and the F/A ratio for the binders in this work must be less than 0.9. In addition, with the exception of the F/A ratios of 1.8 and 2.1 for the RMP100 asphalt binder, all m-values meet the Superpave requirement of not less than 0.3.

With the rapid development of road construction in China, the demand for asphalt fillers is very large. However, since the crushing and ball-milling processes lead to large amounts of environmental pollution, many traditional filler plants have been closed. This has led to a sharp increase in the price of traditional fillers. Considering the drying and transportation costs, the application cost of red mud as filler in asphalt mixture should be lower than traditional fillers. Moreover, the use of red mud can reduce land occupation and environmental pollution. In short, considering the high temperature deformation resistance, low temperature crack resistance, easy construction, and economic factors, it is suggested that the best ratio of RMP, instead of traditional LSP, is 75%. At this point, the optimal F/A of asphalt binder is 1.0.

## Figures and Tables

**Table 1:** Basic performances of LSP and RMP.

Test		LSP	RMP
**Percentage Passing (%)**	0.6 mm	100.0	100.0
	0.3 mm	97.9	98.6
	0.075 mm	91.8	91.3
**Apparent Density (g/cm^3^)**		2.79	2.82

**Table 2:** Chemical composition of LSP and RMP.

Component	LSP (%)	RMP (%)
CaO	46.90	8.76
SiO_2_	17.96	36.01
Fe_2_O_3_	0.51	9.76
Al_2_O_3_	0.46	21.36
TiO_2_	0.035	2.64
Na_2_O	0.081	3.21
MgO	3.64	0.86
K_2_O	0.1	0.77
Others	0.36	2.03
LOI	29.93	14.67

**Table 3:** Studied asphalt binder combinations.

Binder No.	Filler Combination	Binder Type
1	100% LSP + 0% RMP	RMP0
2	75% LSP + 25% RMP	RMP25
3	50% LSP + 50% RMP	RMP50
4	25% LSP + 75% RMP	RMP75
5	0% LSP + 100% RMP	RMP100

**Table 4:** Relationship between the penetration value and F/A ratio.

Binder Type	Model	R^2^ Value
RMP0	y = −13.696x + 68.987	R^2^ = 0.9828
RMP25	y = −13.308x + 61.521	R^2^ = 0.9808
RMP50	y = −13.398x + 62.487	R^2^ = 0.9938
RMP75	y = −15.821x + 64.653	R^2^ = 0.9762
RMP100	y = −23.304x + 72.693	R^2^ = 0.9753

**Table 5:** Relationship between rotational viscosity value and F/A ratio.

Binder Type	Model	R^2^ Value
RMP0	y = 0.3856e^0.908x^	R^2^ = 0.9707
RMP25	y = 0.3616e^1.4201x^	R^2^ = 0.9721
RMP50	y = 0.2986e^1.8517x^	R^2^ = 0.9782
RMP75	y = 0.2779e^2.2624x^	R^2^ = 0.9757
RMP100	y = 0.2486e^2.326x^	R^2^ = 0.9741

**Table 6:** Relationship between complex shear modulus (G*) value and F/A ratio.

Binder Type	Model	R^2^ Value
RMP0	y = 2140.1e^0.5669x^	R^2^ = 0.9704
RMP25	y = 2309.3e^0.7732x^	R^2^ = 0.9894
RMP50	y = 2360.8e^0.9757x^	R^2^ = 0.9871
RMP75	y = 2356.4e^1.0642x^	R^2^ = 0.9804
RMP100	y = 2075.2e^1.1179x^	R^2^ = 0.9768

**Table 7:** Relationship between G*/sin(δ) values and F/A ratio.

Binder Type	Model	R^2^ Value
RMP0	y = 2159.9e^0.5681x^	R^2^ = 0.9704
RMP25	y = 2330.9e^0.7746x^	R^2^ = 0.9895
RMP50	y = 2382.9e^0.9774x^	R^2^ = 0.9871
RMP75	y = 2379.0e^1.0670x^	R^2^ = 0.9805
RMP100	y = 2094.6e^1.1203x^	R^2^ = 0.9769

**Table 8:** Relationship between creep stiffness (S) values and F/A ratio.

Binder Type	Model	R^2^ Value
RMP0	y = 101.41e^0.8691x^	R^2^ = 0.9755
RMP25	y = 87.116e^1.0785x^	R^2^ = 0.9895
RMP50	y = 103.07e^1.0313x^	R^2^ = 0.9827
RMP75	y = 100.27e^1.1348x^	R^2^ = 0.9742
RMP100	y = 99.108e^1.2221x^	R^2^ = 0.9821
